# The rotating magnetocaloric effect as a potential mechanism for natural magnetic senses

**DOI:** 10.1371/journal.pone.0222401

**Published:** 2019-10-01

**Authors:** A. Martin Bell, Jacob T. Robinson

**Affiliations:** 1 Applied Physics Program, Rice University, Houston, Texas, United States of America; 2 Department of Electrical and Computer Engineering, Rice University, Houston, Texas, United States of America; 3 Department of Bioengineering, Rice University, Houston, Texas, United States of America; 4 Department of Neuroscience, Baylor College of Medicine, Houston, Texas, United States of America; University of Salento, ITALY

## Abstract

Many animals are able to sense the earth’s magnetic field, including varieties of arthropods and members of all major vertebrate groups. While the existence of this magnetic sense is widely accepted, the mechanism of action remains unknown. Building from recent work on synthetic magnetoreceptors, we propose a new model for natural magnetosensation based on the rotating magnetocaloric effect (RME), which predicts that heat generated by magnetic nanoparticles may allow animals to detect features of the earth’s magnetic field. Using this model, we identify the conditions for the RME to produce physiological signals in response to the earth’s magnetic field and suggest experiments to distinguish between candidate mechanisms of magnetoreception.

## Introduction

Despite broad scientific consensus that many animals navigate or orient using the earth’s magnetic field, there is no widely accepted biophysical mechanism for magnetoreception [1–7]. Experimental and observational evidence suggests that certain animals can detect three properties of the local magnetic field vector: a polarity or compass sense can detect the polarity of the local field, a direction/inclination sense detects field line direction but gives no polarity information, and a ‘map’ sense detects the intensity of the local field [1]. Any of these modalities (or their combination) could potentially give rise to the migratory and orientation behavior observed in animals.

To explain how animals detect these features of the earth’s magnetic field, scientists have developed hypothetical mechanisms of action that fall into three major categories: electromagnetic induction, magnetic field-dependent chemical reactions, and magnetomechanical force. The induction model proposes that the deflections of charged particles moving in the earth’s field may polarize electrically active cells, resulting in a detectable signal [8]. The next category, chemical-based models, propose that the orientation of the Earth’s magnetic field can alter the the products of chemical reactions [2, 9]. Finally, magnetomechanical models propose that mechanical forces exerted on magnetic materials by external magnetic fields can activate a mechanical response by pulling on ion channels or cell membranes [10, 11]. These concepts are reviewed in detail elsewhere [1, 2, 12].

Despite many efforts, there remains no scientific consensus as to which (if any) of these hypotheses are responsible for natural magnetosensation. The only exception is magnetotactic bacteria, whose ability to align with the earth’s magnetic field is the result of net torque produced by chains of ferromagnets [13]. Elucidating the mechanism for magnetoreception is made even more difficult by recent experimental evidence suggesting different animals may rely on different magnetoreception mechanisms [1, 14–17].

Here we propose a new hypothesis based on recent experiments with synthetic magnetoreceptors [18]. Specifically we argue that heat produced by magnetizing nanoparticles via the magnetocaloric effect could activate nearby thermoreceptors. This magnetocaloric effect was recently proposed as a mechanism to explain synthetic magnetically sensitive ion channels [19] where the alignment of magnetic moments reduces the magnetic entropy (Δ*S*). The reduction of magnetic entropy produces heat (Δ*Q*) according to the thermodynamic relationship: Δ*Q* = −*T*Δ*S* (where *T* is the temperature). In the case of the ferritin nanoparticles used for the previously reported synthetic magnetoreceptors, we calculate this heat to be approximately 6 J/mol, which may be sufficient to produce a physiological response [19]. It should be noted that a physiological response to heat on the order of 6 J/mol is expected only with anomalously low thermal transport at the surface of the magnetic nanoparticle and thermal gradients across the channel protein [19].

While both reduced thermal conductivity and thermal gradients have been reported by several groups [20–23], more work is needed to understand and explain these phenomena.

Here we study whether the magnetocaloric effect could possibly explain natural magnetoreception. Specifically, we ask the following questions:

1. What configuration of magnetic particles and ion channels could create a receptor sensitive to the *direction* of an external magnetic field based on the magnetocaloric effect?
2. How could a population of cells sense the magnetic field direction based on these magnetically sensitive ion channels?
3. Which biogenic magnetic nanoparticles could generate sufficient heat to detect the earth’s magnetic field?
4. What new experiments could determine if animals use the magnetocaloric effect to sense the direction of the earth’s magnetic field?

## Results

### Configurations for a magnetoreceptor based on the rotating magnetocaloric effect

**Fig 1:**
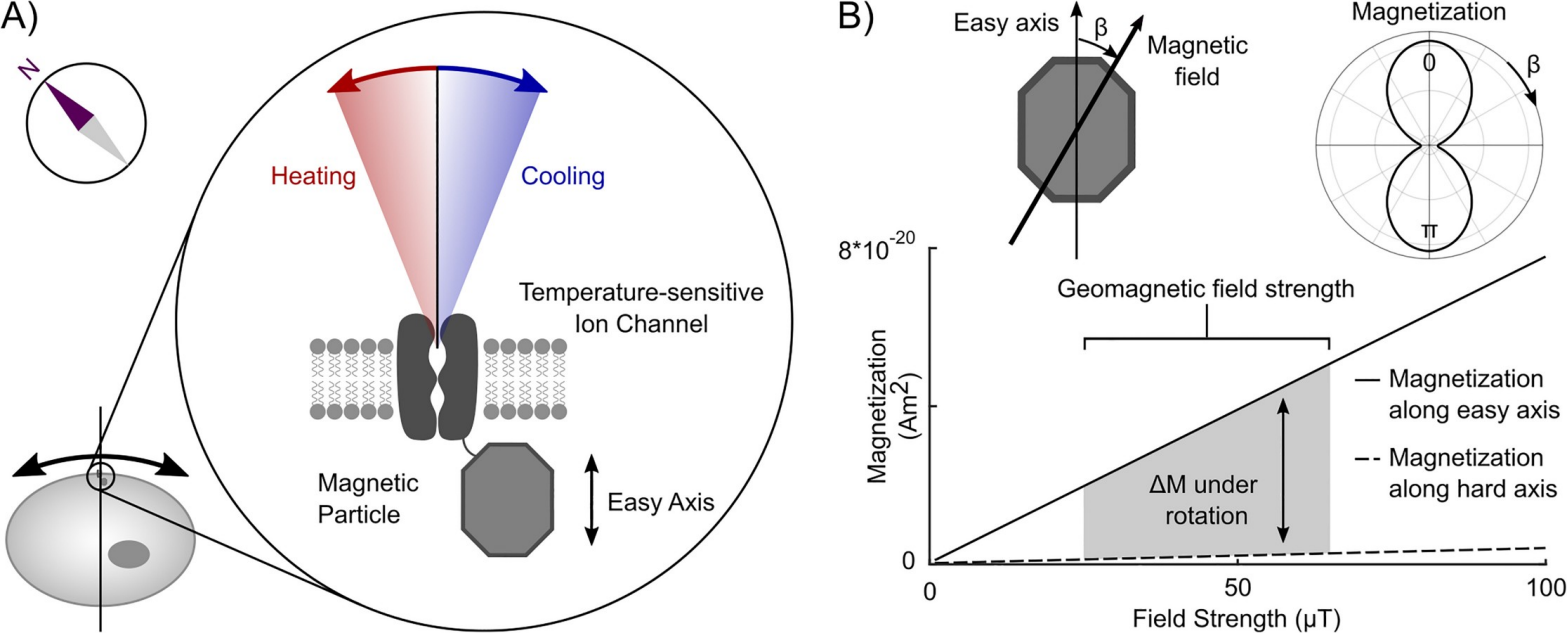
Concept for how the rotating magnetocaloric effect could provide a mechanism for natural magnetosensation. (A) Schematic of a RME-receptor: a magnetically anisotropic particle is bound to a temperature sensitive ion channel. This particle will release or absorb heat energy under rotation in a magnetic field, and this energy could influence the gating of the associated channel. (B) The magnetization of a magnetic nanoparticle is a function of the angle between the easy axis of the particle and the direction of the applied field as shown in the radar plot. Thus rotation will cause the particle magnetization to change, despite no change in the strength of the applied field. For most materials, the easy and hard axis magnetizations are approximately linear for geomagnetic field amplitudes (25-65 μT). As a result, we can calculate the maximum change in magnetization Δ*M* as this difference between the magnetization when the field is along the easy axis (solid line) and hard axis (dotted line). These values are calculated based on 10 nm radius magnetite particles. The gray shaded region shows Δ*M* ∼ 0.4 × 10^−20^ *Am*^2^ for rotation in an Earth-strength magnetic field.

The magnetocaloric effect describes the release or absorption of thermal energy resulting from changes in magnetization. Often these changes in magnetization result from changes in the strength of an applied magnetic field; however, changes in magnetization can also result from rotation within a static field. When a magnetic field is aligned with the energetically favorable “easy” axis of a magnetically anisotropic material, this field produces a larger magnetization compared to when the magnetic field is aligned with the “hard” axis (Fig 1). In this case, rotation of an anisotropic magnetic particle in a static magnetic field produces a change in magnetic entropy. For example, when the easy axis becomes more aligned with the magnetic field, the magnetization is increased. This reduction in the magnetic entropy generates heat. On the other hand, when the easy axis becomes less aligned with the magnetic field, magnetization is decreased, leading to increased magnetic entropy and heat absorption. Thus for particles with magnetic anisotropy, a rotation within a static magnetic field will produce changes in magnetic entropy that, through the magnetocaloric effect, cause the particle to heat or cool. This process is known as the “rotating magnetocaloric effect” (RME) [24].

**Fig 2:**
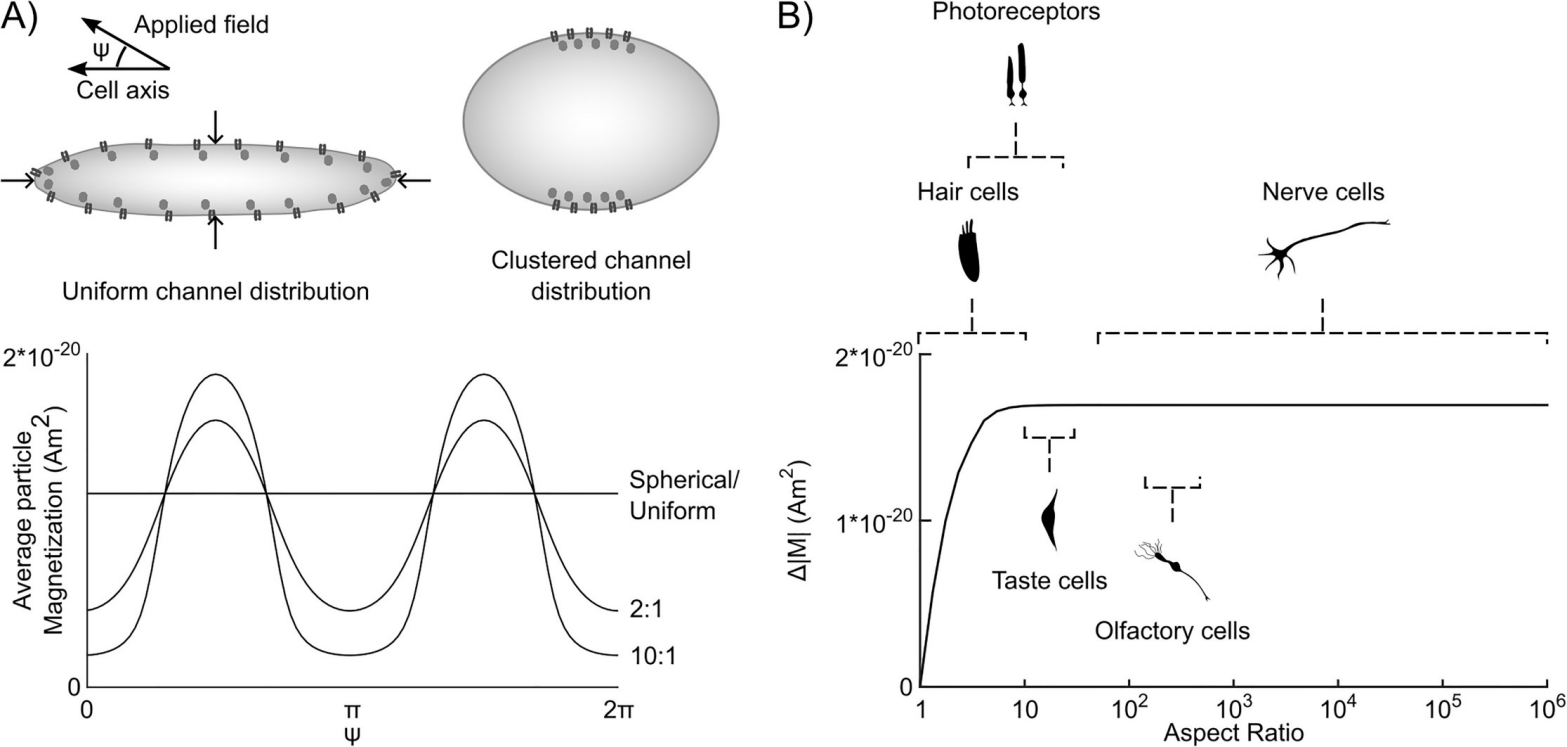
Arrangements of RME-receptors that could produce cells that respond to specific field directions. (A) Two potential arrangements of RME-receptors that would produce a change in average particle magnetization under cell rotation: a high cell aspect ratio with uniformly (or randomly) distributed RME-receptor channels, or a cell of any shape in which the RME-receptor channels cluster together. Either configuration will result in larger numbers of channels oriented along specific axes (along angles $\psi =\frac{\pi }{2}$ in this example), and fewer along others (angles *ψ* = 0, *π* in this example). As a result, the average particle magnetization changes when the cell rotates, which generates or absorbs heat (assuming the easy axis is consistently aligned with the channel pore). Plot shows average magnetization of particles in RME-receptors for prolate spheroidal cells rotating in an Earth-strength magnetic field (with aspect ratios of 1, 2, and 10). Cells with larger aspect ratios will produce greater changes in average particle magnetization under rotation. (B) The maximal change in average channel magnetization is shown as a function of cell aspect ratio for randomly distributed channels with orientation-locked particles. The change in average particle magnetization (which corresponds to the average heat generated or absorbed per particle) increases asymptotically with cell aspect ratio. Cells with aspect ratios greater than ∼ 20 are expected to show near-maximal average magnetocaloric heat generation. Many sensory cells show aspect ratios near or above this number, and thus could be candidate RME-receptive cells. Calculations of magnetization are based on 10 nm radius magnetite particles.

The RME could form the basis of a magnetoreceptor if 1) magnetically anisotropic nanoparticles were associated with temperature sensitive ion channels; and 2) the particle’s easy axis was consistently aligned at a specific angle with respect to the cell membrane (Fig 1). In this case, when the animal’s movements align the easy axis to the earth’s magnetic field, heat produced by the RME could activate the associated thermoreceptor. If the orientation between the easy axis and the thermoreceptor was random, when the animal moved an equal number of channels would heat and cool resulting in no net effect. If, however, the nanoparticle orientation can be controlled relative to the channel, animal movement could result in net heating or cooling of the ensemble of thermoreceptors in a given cell, allowing it to sense the direction of a static magnetic field. Thus, for the effects of many receptors to be additive, the easy axis of the particle must have a stereotyped orientation relative to the ion channel or cell membrane (Fig 2).

Several examples in nature show that magnetic nanoparticles in living organisms can be specifically oriented relative to proteins based on the particle shape and/or specific crystal faces that correspond with the easy or hard axes. For example in magnetotactic bacteria the formation and morphology of the biogenic magnetic crystals that give the bacteria their orientation ability are tightly regulated [13, 25]. This precision comes from proteins which initiate and control magnetic particle formation. Several of these proteins, such as Mms5, Mms6, Mms7, and Mms13 are capable of binding to the crystal surfaces [25, 26], and other mineral-associated proteins such as mamC and osteopontin have even been shown to bind preferentially to specific crystal faces [27, 28]. Such preferential binding could result in the type of stereotyped orientation required for a RME based magnetoreceptor.

Hereafter, we will refer to the configuration of an anisotropic magnetic particle aligned with a thermoreceptor as an “RME-receptor”.

### A hypothetical magnetoreceptor cell

Based on the RME-receptors described above, we propose two candidate “magnetoreceptor cells” that would depolarize based on reorientation within a static magnetic field. The two candidate magnetoreceptor cells we propose are: 1) a cell with an elongated morphology that expresses RME-receptors uniformly on the cell membrane; or 2) a cell of any morphology that expresses RME-receptors non-uniformly on the cell membrane (Fig 2). A spherical cell, with channels uniformly distributed on the cell membrane would have an equal number of channels activated and inactivated as the magnetic field direction changes. In other words, the total magnetization of the RME-receptors would be the same for all orientations of the magnetic field and the cell response would show no selectivity for any particular magnetic field direction. Thus, for a cell to encode a specific magnetic field direction (or change in direction), the total magnetization of all the RME-magnetoreceptors must be a function of the cell’s orientation with respect to the magnetic field.

We find in the case of uniform channel distribution, there is little increase in the cell’s sensitivity to the magnetic field angle for aspect ratios greater than 20. Many varieties of sensory cells have aspect ratios near or above 20 [29–33], making them potential candidate magnetoreceptors (Fig 2). Neurons are extreme examples of elongated cells and would be a natural choice for magnetoreceptor cells. In addition, neurons and many other cells are also known to traffic ion channels to specific compartments on the cellular membrane, like the axon hillock [34, 35]. This non-uniform channel distribution could also lead to sensitivity to magnetic field direction.

### Candidate magnetic particles for RME-magnetoreceptors

For a particle to act as a magnetosensor, the magnetic moment must be able to realign at the timescale of animal movement (or faster). This realignment of the magnetic domain occurs via one of two relaxation processes: Brownian relaxation, where the entire particle rotates while the magnetic moment remains fixed relative to the particle axis; and Néel relaxation, where the magnetic moment reorients without rotating the particle.

To establish a parameter space of potential nanoparticle materials and sizes, we examined the relaxation times for a variety of nanoparticle sizes and biogenic magnetic materials. To establish this parameter space, we used the time constant associated with Néel relaxation to identify combinations of magnetic moment and anisotropy energy for which the particle relaxes fast enough for use in a magnetic sense (See Methods).

The time constant associated with Brownian relaxation is given by $\tau_{B}=\frac{3\eta V_{H}}{k_{B}T}$, where *η* is the viscosity of the surrounding liquid, *V*_*H*_ is the hydrodynamic volume of the particle, *k*_*B*_ is the Boltzmann constant, and *T* is temperature [36]. For particles with high anisotropy, this time constant is often much faster than the Néel relaxation time constant, *τ*_*N*_, and might be expected to determine the relaxation rate. However in the case of particles that are not permitted to physically rotate (for example particles immobilized through binding to membrane proteins), Brownian relaxation is prohibited, and magnetic relaxation may only occur through Néel relaxation.

**Fig 3:**
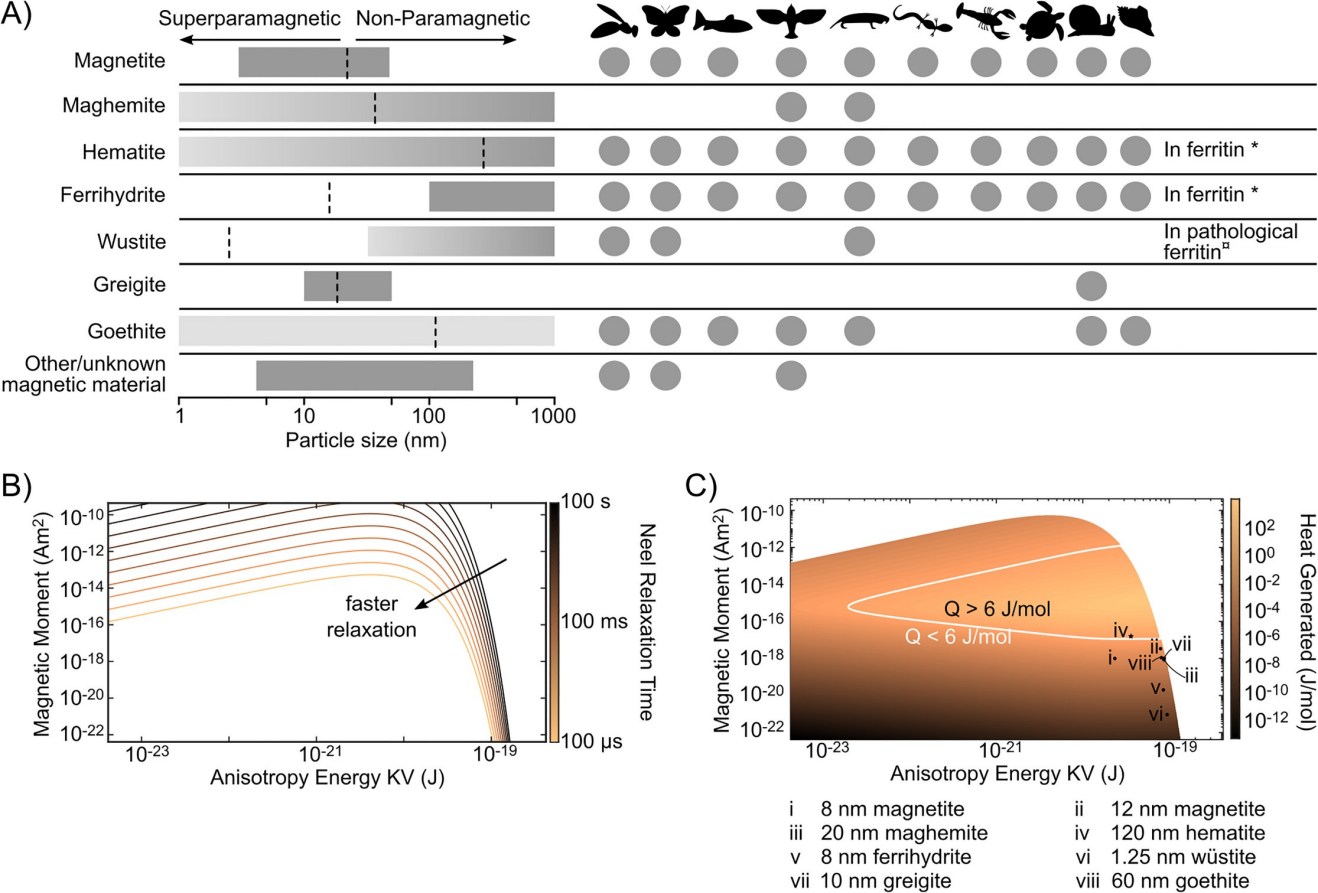
Experimentally observed biogenic magnetic particles include good candidates for magnetocaloric heat changes. (A) Rows show materials found to be present in biogenic nanoparticles. Grey bars indicate sizes found in literature [10, 12, 15, 39–41], with lighter gray representing uncertainty about limits (for example, goethite particles are found in Columba livia pigeons, but the particle size is unconfirmed). Black dashed lines represent the boundary (critical diameter) between the superparamagnetic and non-superparamagnetic domains for spherical particles at 300K according to Néel relaxation theory. Grey circles indicate that nanoparticles of the materials indicated on the left have been identified in one or more members of the order indicated. Animal orders shown are: Hymenoptera, Lepidoptera, Salmoniformes, Columbiformes and Passeriformes, Rodentia, Caudata, Decapoda, Testudines, Panpulmonata, and Patellogastropoda. (B) The Néel relaxation rate of a particle can be predicted via Néel relaxation theory using the magnetic moment and anisotropy constant of the particle. Contour lines show consistent Néel relaxation times as a function of magnetic moment and anisotropy energies. Contours range from 100 microseconds to 100 seconds. Assuming the Néel relaxation must be faster than the time of animal reorientation constrains the parameter space of allowed superparamagnetic nanoparticles. For particles with magnetic moments < 0.1*fAm*^2^ a 1,000,000x increase in the choice of relaxation time threshold results in an increase in maximum particle anisotropy of <10x. (C) Maximum heat generated under rotation as a function of magnetic moment and particle anisotropy energy, for particles with relaxation values below 100 milliseconds. The white line shows the threshold for particles that are expected to produce at least as much heat under rotation (≥ 6 J/mol) as is expected from the synthetic magnetosensor Magneto 2.0 under application of a 275 mT field. Black icons represent particles of materials whose presence has been established in animals with magnetic senses. Of these, hematite (black star) has been found in sizes capable of generating greater than 6 J/mol. Black circles show other magnetic materials found in animals with magnetosensory abilities, but whose material properties and size distributions are less suited for use in RME-receptors. Materials: i) 8 nm radius magnetite, ii) 12 nm magnetite, iii) 20 nm maghemite, iv) 120 nm hematite, v) 8 nm ferrihydrite, vi) 1.25 nm wüstite, vii) 10 nm greigite, viii) 60 nm goethite. Heat generation values assume 25 μT field, near the lower limit for the range of observed GMF field strengths and use published values of material saturation magnetization strengths and anisotropy constants, and a rotation from aligned with the hard axis to aligned with the easy axis [47–59]. *[39, 60–62] ¤[39].

The requirement for *τ*_*N*_ to be smaller than the timescale of animal reorientation provides an upper limit to both magnetic moment and anisotropy energy (Fig 3b). In this work we have used 100 ms, however the choice of minimum *τ*_*N*_ has minimal effect on the parameter space. As can be seen in Fig 3, changes in *τ*_*N*_ by 4 orders of magnitude would approximately double the allowed anisotropy constant for biogenic particles.

### Nanoparticle heat generation using the rotating magnetocaloric effect

To determine the lower limit for anisotropy and moment, we examined the anticipated heat generation resulting from nanoparticles under rotation in earth-strength magnetic fields. There are three key steps in this process: determining the magnetization of the particle throughout rotation, calculating the resulting entropy change, and then finding the anticipated change in thermal energy.

We calculated the magnetization and magnetization change using the Jiles-Atherton model [37], calculated the change in entropy from magnetization along a particular orientation according to $\Delta S=\int_{0}^{B}{\left(\frac{\partial M}{\partial T}\right)}_{B}dB$, and the change in thermal energy by taking the difference in entropy along the starting and ending axes: $Q=T\Delta S_{\beta_{f}}-T\Delta S_{\beta_{0}}$ [38]. (See Methods)

For the rotating magnetocaloric effect to be responsible for a magnetic sense, the resulting heat generation must be sufficient to affect ion channel activity. In this work, we have chosen 6 J/mol as the minimum heat needed to affect cell activity because this is the expected heat generated by the ferritin component of magneto2.0 and MagM8 under the magnetization conditions described in Duret, Polali, et al. [19]. We see in Fig 3 that there are indeed materials that can generate energy changes near this threshold while maintaining Néel relaxation times shorter than 100 ms. This set of candidate particles includes several particle sizes that have been directly observed in animals with magnetic senses, as well as particles of other biogenic materials (Fig 3) [10, 12, 15, 39–41]. From the parameter space established by the relaxation rate (Fig 3), we can see that the best candidate nanoparticles for heat generation by the RME are particles with large magnetic moments and large particle anisotropy. Especially promising candidates therefore include the iron oxides hematite and magnetite. Hematite is able to form large, single domain superparamagnetic particles [42, 43] and is predicted produce upwards of ten times more heat than the 6J/mol produced in Magneto2.0 reported previously [19]. Additionally, magnetite, while being just shy of this 6J/mol, may also be a viable transducer. Paired with TRPV4 and following same model described previously [19], we would expect ∼10^4^ RME sensors per cell to allow consistent responses from magnetite. For comparison, TRP channel expression levels in nature have been found to be as high as ∼10^7^ channels per cell [44].

One should note that nonidealities resulting from particle shape effects, surface imperfections, or material-specific crystal structures can affect the precise heat generation under rotation. [45] Effects from crystal irregularities or from particle shape can either increase or decrease particle anisotropy compared to the bulk values for that material. Additionally, the effect of uncompensated spins at the surfaces of particles can act to increase or decrease the magnetic moment of a particle from the moment predicted by its bulk saturation magnetization. Finally, these heat generation values use a uniaxial model of anisotropy, and additional axes of anisotropy can change the magnetic energy landscape by changing the form of the anisotropy energy term. For these reasons, particles could produce greater heat release than expected, if the particles structure yielded anisotropy energies and/or magnetic moments different than those predicted by bulk measurements [46].

## Methods

Calculations were performed using custom MATLAB scripts, which we have made available on the Open Science Framework, using values for material properties and particle sizes from literature [39, 47–62].

### Calculating material magnetization

To determine the magnetization of a particle at steady state as a function of applied magnetic field, crystal/field orientation, and material properties, we wrote a MATLAB script based on the Jiles-Atherton model with Ramesh extension [37, 63, 64]:

$$\begin{array}{c} <M>=M_{s}\frac{\int_{0}^{\pi }e^{\frac{-E(\theta )}{k_{B}T}}sin\left(\theta \right)cos\left(\theta \right)d\theta }{\int_{0}^{\pi }e^{\frac{-E(\theta )}{k_{B}T}}sin\left(\theta \right)d\theta } \end{array}$$

Where *M*_*s*_ is the particle magnetic moment at saturation magnetization, and *E*(*θ*) is the energy of orienting the magnetic moment along angle *θ*, given (in the case of uniaxial anisotropy) by $E\left(\theta \right)=\frac{1}{2}\left(-2\mu Bcos\left(\theta \right)+KV\left(sin^{2}\left(\beta -\theta \right)+sin^{2}\left(\beta +\theta \right)\right)\right)$, where *K* is the material anisotropy constant, *V* is the particle volume, *μ* is the magnetic moment, *B* is the applied field, and *β* is the angle between the applied field and particle easy axis. We assumed spherical particles with uniaxial anisotropy, with values for saturation magnetization and anisotropy constant from literature.

### Calculating relaxation time

The time constant of Néel relaxation is given by $\tau_{N}=\tau_{0}e^{\frac{KV}{k_{B}T}}$, where *τ*_0_ is the “attempt time” or “event time”, given by $\tau_{0}=\frac{\mu }{2\mu_{0}\gamma \alpha K}$. Here, *γ* is the electron gyromagnetic ratio and *α* is a magnetic damping constant that varies from 0.01 to 1 [65]. For these calculations, we set *α* to 1, which is common for magnetic nanoparticles [65]. Lower values of *α* would increase the value of *τ*_0_ and thus *τ*_*N*_.

The time required for Néel relaxation depends on the magnetic anisotropy energy of the particle, *KV*, where *K* is the anisotropy constant of the material, *V* is particle volume. The dependence of relaxation time on the anisotropy energy results from its influence on the energy barrier along the particle hard plane through which the magnetic moment to must rotate in order to randomize the distribution of particle magnetic moments.

### Calculating heat generated by the rotating magnetocaloric effect

The heat generation *Q* from the rotating magnetocaloric effect for a uniaxially anisotropic particle from angle *β*_0_ to angle *β*_*f*_ is given by the difference in the heat produced by magnetization along those axes: $Q=T[\int_{0}^{B_{f}}{\left(\frac{\partial M_{\beta_{f}}}{\partial T}\right)}_{B}dB-\int_{0}^{B_{f}}{\left(\frac{\partial M_{\beta_{0}}}{\partial T}\right)}_{B}dB]$ [38]. To generate the heatmap in Fig 3, we use these equations to calculate the change in magnetization and the heat generated under rotation. Because the relationship between magnetic moment and heat generated is log-linear below saturation (Fig B in S1 File), we can approximate the heat generated by multiplying the difference in magnetization along the starting and ending orientations by a scaling factor *Q* ≈ *ξ*(*B*, *KV*)Δ*M*. This approximation is accurate to within 1% for all particles plotted when compared to the complete expression (Fig B in S1 File).

### Calculating magnetization effects at cellular level

To calculate the orientation selectivity of a RME cell, we modeled cells as prolate spheroids, and calculated the mean magnetization of all particles as a function of magnetic field orientation (assuming a fixed relationship of the particle easy axis with the ion channel pore axis). To calculate the effect of non-uniform channel distribution we weighted the integral across the cell surface by channel distribution density function *ρ*(*ϕ*, *θ*).

## Discussion

As we have described, a magnetocaloric particle will generate or absorb heat due to changes in magnetization, which can come from either changes in orientation relative to an external field, or from changes in the strength of that field. Our calculations predict that this mechanism could potentially give rise to a magnetic sense able to detect rotation in an earth-strength magnetic field. In this section, we look to experimental and observational findings around the physiology and behavior of magnetosensory animals to explore the feasibility of the magnetocaloric model. We then propose a new category of experiment to probe the mechanisms behind natural magnetosensation.

### Candidate RME-receptor channels

One of the challenges associated with identifying the locus of a magnetic sense is the difficulty in measuring the distribution of biogenic magnetic nanoparticles. These particles are small, hard to resolve, and can be altered or dissolved by fixatives commonly used in sample preparation [1, 66]. Additionally, iron oxides are common in nature, which presents a risk of false positives, as with the identification of iron-rich cells in pigeons that were later shown to be macrophages [67]. Iron oxides are also common in the lab, which means that sample processing protocols must be extremely careful not to introduce new materials. These difficulties suggest that in addition to searching for magnetic nanoparticles responsible for magnetosensation, one should also conduct behavioral experiments to provide clues about the mechanism of action.

Alternatively, one may search for thermoreceptors that may form the basis of an RME-receptor. The RME model of magnetoception predicts that the thermoceptors most effective for a magnetic sense would have a large entropy of gating and a sensitivity peak near the body temperature of the animal. Several channels display these properties, including TREK-1, TRAAK, TRPV3, TRPV4, TRPM3, and TRPM5, making them good candidates. For birds, TRPV1 may also be responsible, while for fish and reptiles TRPM4 or even TRPM8 or TRPC5 may contribute to a magnetic sense. [68–77]]. Interestingly, TRPV4, the channel upon which the synthetic magnetosensor Magneto2.0 is based, has a particularly high entropy of gating at 1496 $\frac{J}{molK}$ [19].

### A magnetic sense based on magnetocaloric receptor cells

According to our RME hypothesis, an animal rotating in a magnetic field would experience increases or decreases in the activity of their magnetically sensitive cells, when their rotation caused the easy directions of their magnetic sensors to align more or less with the external field. Movements that result in increased alignment would increase cell activity, while movements that decreased alignment between easy directions and the magnetic field would decrease cell activity. By turning, animals could therefore ensure they were maintaining the desired orientation within the field. This type of cellular response could produce a standalone magnetic sense, processed in a similar manner to other senses, of which the animal is actively aware. But it is also possible that these cells act to modulate other senses, piggybacking on the existing architecture to deliver magnetic information, as has been proposed for avian magnetosensation [9, 78, 79].

A magnetocaloric magnetic sense following this model would be expected to be an inclination/direction sense and not a polarity sense. That is, capable of detecting the direction, but not polarity or strength, of the external magnetic field.

To obtain information about latitude, landmarks, or to distinguish north/south, animals could compare this information about the magnetic field strength with information from their other senses, for example the vector of gravitational force. Similar approaches have been proposed for the polarity insensitive chemical hypothesis of magnetosensation [1, 78].

An intensity sense or a polarity sense both might be possible under an RME model, but add additional constraints to either the protein distribution or protein regulation processes in the cell. An “absolute” field intensity sense would require a very tightly regulated protein expression setting the cell’s dynamic range, and exchange bias (for example resulting from surface defects or nearby magnetic material such as other magnetic crystals) could give rise to a polarity sense. As a result of the additional complications associated with an intensity sense or a polarity sense, we view a directionally sensitive magnetosensor cell as being the most probable incarnation of a magnetosensor cell based on RME-receptors.

### Experimental support for the rotating magnetocaloric effect as a mechanism for magnetoreception

Because each of the models of magnetosensation make slightly different predictions about how a magnetoreceptive cell will respond to changes in the external magnetic field, researchers have used external fields to experimentally probe the mechanisms underlying magnetosensation. Experiments intended to differentiate between major models of magnetosensation include the application of strong magnetic pulses, weak radio frequency magnetic fields, sudden field polarity reversals, or different lighting conditions. These parameters are intended to be detectable by one or more mechanisms, and undetectable by others, and thus can provide insight into the type (or types) of magnetic sense in the animal being investigated. Here we introduce some of these experimental results and explain how they support or oppose each of the chemical, mechanical, and magnetocaloric models of magnetosensation.

#### Strong, brief magnetic field pulses

Prolonged disorientation following a brief, strong (>0.1 T) magnetic field pulse most strongly supports a magnetomechanical model of magnetosensation based on permanently magnetized particles. The rationale behind this test is that large, permanently magnetized particles can potentially be remagnetized along the axis of the strong applied pulse which is applied before the particles have time to physically rotate. Thus, animals relying on a magnetomechanical sense should therefore show deflected orientation after the applied pulse [[10, 80, 81]]. Prolonged disorientation could then result as the particles relaxed to their original energetically favorable magnetization states, or were replaced. It is also hypothesized that clusters of superparamagnetic particles may be temporarily disrupted by the pulse [82], impairing the animal’s magnetosensory capability as the clusters are reformed.

On the other hand, the chemical magnetosensation model is not expected to show lasting effects from a strong magnetic field pulse [15, 82]. The chemical model is a temporal effect that depends on the presence of the field throughout the reaction. While the organism may detect the pulse, it’s unlikely to result in prolonged disorientation (Fig D in S1 File). Similarly, under our magnetocaloric model, during a strong magnetic pulse any particle not at saturation would experience increased magnetization and the associated RME-receptors would increase in activity, leading to immediate disorientation, but the RME-receptors should return to normal function after the pulse.

Experiments show that strong magnetic field pulses do indeed disrupt or otherwise affect magnetic orientation in birds [81, 83–85] and turtles [86], though the effect appears to be age-dependent [80]. For example, in Australian Silvereyes, a species of migratory bird, exposure to a brief (4-5 millisecond) magnetic pulse of 0.5 T resulted in 4 days of deflected orientation behavior, as measured by placing the bird in Emlen funnels and recording the direction of attempted movement around sunset. After the initial deflection, preferred orientation varied in direction for several days before returning to the natural southward migratory direction. [87] Interestingly, this effect is not observed in juvenile birds of the same species [88], whose orientation behavior over timescales of several days appears weakened by strong magnetic pulses but not lost. Migration tracking studies on other migratory birds such as European robins show similar age-dependence [80], but show only loss of orientation, not deflection. Pulse experiments also affect some aspects of the magnetic sense of spiny lobsters [41], sea turtles [86], and mammals including bats [6].

Initially these results seem to lend support to a magnetomechanical model; however, under both the magnetocaloric and chemical magnetosensation models, as well as the magnetomechanical model, simple disorientation or loss of magnetic orientation over the course of hours could also result from changes in protein expression following a magnetic pulse. Because the geomagnetic field varies in strength spatially (from ∼ 25 − 65 *μT*) [89], it seems probable that the magnetosensory proteins used by migratory species are regulated to optimize sensitivity to the local magnetic field. In this case, the strong and synchronous activation of the magnetoreceptors (as would result from strong magnetic pulses) could result in desensitization or downregulation of the proteins involved, resulting in a temporary loss of sensitivity, potentially through similar mechanisms as seen in agonist- and calcium- induced desensitization of TRPV1 [90] which can trigger desensitization for periods lasting minutes to days. If this physiologically anomalous stimulation causes the cell to downregulate the proteins involved in magnetosensation, the animal could show disorientation on the timescales of regulation of gene expression. Thus the protein expression changes associated with rapid and lasting desensitization following application of strong magnetic pulses may provide insight into the mechanism involved in the magnetic sense of a given animal.

It should also be noted that in some cases, such as with transcranial magnetic stimulation (TMS), short, high-amplitude magnetic fields (>1 T fields, with field strength ramp ∼10,000 T/s is typical for clinical TMS) can stimulate neurons directly by inductive currents [91]. Experiments intended to probe the mechanism of a natural magnetic sense must be designed to avoid inducing strong eddy currents which could produce this kind of direct stimulation.

#### Radio frequency oscillating magnetic fields (and reaction yield detected magnetic resonance)

Another experiment proposed as a potential means of identifying the mechanism underlying the natural magnetic sense is the application of a radio frequency alternating magnetic field (RFAMF). Disorientation under weak RFAMF fields most strongly supports the chemical and magnetocaloric models of magnetosensation. The radical pair model of chemical magnetosensation depends upon the interactions between the electron spin and magnetic fields from both external sources and the rest of the molecule through hyperfine interactions [2]. The effect of the hyperfine interactions has a set of characteristic interconversion frequencies, and oscillating magnetic fields applied at those frequencies (typically MHz range) are expected to disrupt the magnetic sense, with larger effects seen in molecules exhibiting fewer hyperfine interactions [9, 92, 93]. The weak fields used in these magnetosensation experiments are not expected to generate notable heat via the magnetocaloric effect. Under an alternating magnetic field, however, there are 3 additional mechanisms of heat release in magnetic materials which could affect cells that sense magnetic fields via a magnetocaloric mechanism: relaxation losses, hysteresis losses, and eddy currents resulting in Joule heating [94]. At these particle scales, joule heating from eddy currents will be negligible [95], but relaxation and hysteresis losses may produce sufficient heating to affect the activity of heat sensitive RME-receptors for frequencies $f\approx \frac{1}{\tau_{N}}$ (Fig E in S1 File).

Magnetoreceptors that rely on magnetomechanical effects are not expected to be affected by RFAMFs, because the relaxation time of ferromagnetic particles is much longer than the magnetic field cycle time (∼1*μs*). As a result, the particles do not have time to move significantly in the timescale of field oscillations. Superparamagnetic and paramagnetic mechanisms could still produce an effect in an oscillating field, as the moments may have time to flip and produce small attraction/repulsion forces, but the weak field strengths used in these experiments mean this effect would be undetectable (discussed in S1 File).

In experiments, oscillating magnetic fields have been shown to disrupt magnetic orientation in birds [92, 96–98]. Though these experimental results have not shown the frequency dependence predicted by theory, the results are still broadly consistent with the predictions of the chemical magnetosensation model [2, 9], in that RFAMFs produce disorientation. This effect is also consistent with the behavior predicted by the magnetocaloric model. Given the field strengths and frequencies used in these experiments, heat produced by relaxation losses in RFAMFs can be greater than 6 J/mol per second (Fig E in S1 File). This RFAMF heat generation may be sufficient to affect heat-sensitive channels, and could result in the types of disorientation seen in experiments.

Additionally, experiments in European Robins showed an angular dependence to the effect of RFAMFs. These birds were able to orient when the RFAMF was aligned with the geomagnetic field, but not when it was vertical or had the horizontal component of the vector flipped [92]. Under a magnetocaloric mechanism, this could possibly occur because the direction of maximum magnetization is the same as the direction of maximum magnetic heating. Thus the the animal would still feel “more aligned” when turning towards alignment with the geomagnetic field. Interestingly, the stimulation parameters used in these experiments (7 MHz, 0.47 μT) are predicted (using linear response theory) to produce heating of nearly 34 kW/mol in 10 nm radius magnetite particles. This means that for these particles, the small amplitude RFAMF would produce more heat through hysteresis in less than 5 milliseconds than the RME would produce under rotation.

#### Polarity reversals

Another experiment used to probe the mechanism of a magnetic sense is to rapidly reverse the direction of the external field. Sensitivity to sudden polarity reversals most strongly supports the magnetomechanical and magnetocaloric models, while sustained field polarity detection most strongly supports a magnetomechanical sense based on single domain particles. In the magnetomechanical model, prolonged effects could result from permanently magnetized single domain particles [10], which would apply torque flipped 180 degrees under an inverted field.

A magnetomechanical sense based upon superparamagnetic or paramagnetic materials would be insensitive to polarity [11], but depending on the rate of change could potentially detect the moment of the field reversal. Similarly, the model we have presented of a magnetocaloric sense based on uniaxially anisotropic superparamagnetic particles is polarity agnostic but it is likely that a magnetocaloric sense would detect the sudden changes in field strength or direction accompanying the moment of field reversal (Fig D in S1 File). The chemical model of magnetosensation does not, at present, have a way to explain a static polarity sense [9]. The product yields depend on the precession rate of the radical spins, which is independent of field polarity, but again the moment of change might be detected (Fig D in S1 File).

Some animals, including salmon, spiny lobsters, and mole rats, show sensitivity to the polarity of magnetic fields, orienting differently when the field reverses direction or showing an ability to detect the moment of field reversal [99–101].

As discussed earlier, exchange bias and consistently nonuniform RME-receptor distribution within cells could potentially permit sustained field polarity detection even under the magnetocaloric model. However, a polarity sense, if it can be appropriately separated from the moment of field reversal, most strongly supports a magnetomechanical mechanism.

#### Light dependence

Finally, due to the explicit light dependence of the cryptochrome hypothesis, the most prevalent chemical model of magnetosensation, a dependence of magnetosensory ability on lighting conditions most strongly supports a chemical model of magnetosensation. This is because the radical pair model of chemical magnetosensation requires incident photons to generate the radicals that are key to the orientation dependence of the product yields [9]. To our knowledge, there is no model of mechanical magnetosensation that would have an explicit light dependence, as the attraction, repulsion, or rotation of magnetic particles should not depend on lighting conditions. Similarly, we do not propose any explicit light dependence for the magnetocaloric model.

The magnetic orientation of some magnetosensitive animals, including many birds and salamanders, shows dependence upon light, with orientation affected by both wavelength and intensity [85, 88, 102–105]. For example, in European Robins, longer wavelengths and lower intensities of light decreased the orientation preference of the animals [102].

However, there are at least two ways that animals using a light-insensitive magnetic sense could behave differently under different lighting conditions. 1) The mechanism of magnetic sense transduction may act by modulating the information provided by a different sensory pathway. For example, the magnetic sense may depend on vision in some animals; 2) light could affect motivation of animals to orient themselves [12, 102]. As a result, while the chemical model of magnetosensation does have a proposed mechanism for direct light dependence, the presence of a light-dependent magnetic sense is insufficient evidence to rule out other models of magnetosensation. On the other hand, a light-independent magnetic sense is sufficient evidence to rule out a magnetic sense based on radical pairs created through photon absorption. We hope that experiments at a cellular level can help determine the origin of light-dependent orientation, as well as explain its absence in other animals with a magnetic sense (notably in marine and burrowing animals).

#### Mixed stimuli tests

As we have seen, due to the large number of confounding factors that can affect animal behavior, experiments varying a single parameter have been insufficient to clearly identify the mechanism of magnetosensation in animals. As a result, researchers have sometimes looked to combine stimuli to probe the magnetic sense. For example, turtles exposed to a series of strong (40 mT) magnetic pulses immediately before being allowed to swim for 60 min in light followed by 60 min in darkness maintained their heading in light but showed no consistent orientation in darkness, while control turtles tended to maintain a similar heading in darkness as in light [86]. This example shows seemingly conflicting results: light dependent orientation suggests chemical magnetosensation has a role, but then the control turtles should be unable to orient in darkness; similarly, the effect of strong pulses suggests a role for a magnetomechanical sense involving single domain particles. However, light can affect animals in many ways that aren’t magnetic, making it difficult to tell if light is truly integral to a magnetic sense.

### Validating the RME hypothesis: What new experiments would better test this mechanism of action?

To better differentiate between competing hypotheses for magnetoreception, we propose two additional categories of experiment:

#### Changing amplitudes of radio frequency alternating magnetic fields

The heat generation of particles under RFAMFs, and resulting effect on channels responsible for a magnetic sense in the magnetocaloric hypothesis, is expected to increase with increasing frequency. However due to the difficulties associated with predicting the effect under a chemical model, an observed increase may not be sufficient to confirm a magnetocaloric mechanism. As discussed above, chemical magnetosensation could be disrupted by alternating magnetic fields with frequencies matching the frequencies of state interconversion. Unfortunately, predicting how the effect will scale with frequency depends on detailed knowledge of the structure of the radical pair molecules [9].

A parameter which may give more useful information than frequency is the amplitude of the applied alternating magnetic field. The chemical, magnetomechanical, and magnetocaloric models of magnetosensation should all scale differently with the amplitude of RFAMFs. The effect of alternating magnetic fields resulting from a magnetomechanical mechanism is predicted to decrease with increasing frequency, as the force delivered to the magnetic particles acts for shorter and shorter amounts of time, resulting in no net force on the channel during the timescale of a single channel gating (discussed in S1 File). Additionally, the magnetomechanical model shows a strong field-strength dependence, so weak, high frequency fields are expected to have a negligible effect on a magnetomechanical sensor.

RFAMF experiments performed at fields weaker than the GMF are expected to show an effect under the radical pair and magnetocaloric models of magnetosensation, so effects from weak RFAMF fields are sufficient to show the presence of a non-mechanical mechanism. The power delivered by magnetic heating scales with the square of the amplitude of the AMF, but the effect of chemical magnetosensation is expected to show peaks and valleys before reaching an asymptote [106]. Behaviorally these may be difficult to distinguish, but at a cellular level the differences should be apparent. Additionally, since the chemical model relies on light, disorientation in animals able to orient in the absence of light would provide strong support for the magnetocaloric hypothesis.

#### Rotation at high fields

Because the magnetocaloric effect depends on a change in magnetization, changes in saturation-strength fields will produce small changes in magnetization and thus little heating or cooling. As a result, RME-receptor cells will become less sensitive to magnetic fields at very high field strengths (Fig F in S1 File). On the other hand, the radical pair and magnetomechanical models predict no such decrease in sensitivity. As a result, a loss of an animal’s magnetosensory abilities at high fields would support the magnetocaloric hypothesis.

## Conclusion

We have explored how the magnetocaloric model of magnetosensation might give rise to a magnetic sense in nature, and identified candidate biogenic nanoparticles. Our results suggest that the magnetocaloric mechanism could potentially give rise to a magnetic sense in animals that would permit orientation in the earth’s magnetic field. The tests and candidate materials outlined here will inform experiments to identify magnetically sensitive cells and animals that may make use of the rotating magnetocaloric effect to sense magnetic fields. Additionally, by designing around the principles underlying this magnetocaloric model of magnetosensation, our findings suggest that it may be possible to develop synthetic channels sensitive to sub millitesla magnetic fields, or even to develop directionally sensitive synthetic magnetoceptors.

## Supporting information

S1 File Discussion of nuances to magnetosensory hypotheses. This supplement discusses our rationale for using the model of magnetocaloric heat generation used in this work, explores how even “polarity insensitive” mechanisms may detect changes in polarity of applied fields, discusses the specifics of the effects of RFAMFs on magnetocaloric or magnetomechanical magnetoreceptors, and models the effect on magnetocaloric magnetosensors of field changes and rotation at high field strengths.
(PDF)
