# Supplementary material for: The rotating magnetocaloric effect as a potential mechanism for natural magnetic senses
Source: PLoS One. 2019 Oct 1;14(10):e0222401. doi: 10.1371/journal.pone.0222401 (PMC6773214; doi:10.1371/journal.pone.0222401)
Supplement: S1 File — This supplement discusses our rationale for using the model of magnetocaloric heat generation used in this work, explores how even “polarity insensitive” mechanisms may detect changes in polarity of applied fields, discusses the specifics of the effects of RFAMFs on magnetocaloric or magnetomechanical magnetoreceptors, and models the effect on magnetocaloric magnetosensors of field changes and rotation at high field strengths. (PDF) [file pone.0222401.s001.pdf]

Supplementary Information

Calculating heat generation from the rotating magnetocaloric effect

The approach we use to calculate the heat generated by the rotating magnetocaloric effect is based on the Maxwell relation $\frac{\partial S}{\partial B} = \frac{\partial M}{\partial T}$. This can then be rearranged and integrated to find the change in entropy resulting from a change in the magnetic field: $\Delta S = \int_{B_0}^{B_f} (\frac{\partial M(B,\theta)}{\partial T})_B dB$ [1].

At B=0, there is no change in magnetization under rotation ($M(B = 0, \theta) = M_0$ for all θ). As a result, we can rotate the particle freely under zero applied field with no change in magnetic entropy. Because entropy is a state variable and thus path independent, this means that we can calculate the heat generated under rotation at fixed applied field amplitude by finding the difference in the heat generated under magnetization along the starting and ending orientations. $\Delta S = \int_{0}^{B_f} (\frac{\partial M(B,\theta_f)}{\partial T})_B dB - \int_{0}^{B_0} (\frac{\partial M(B,\theta_0)}{\partial T})_B dB$ (Fig A).

To calculate heat generation in this way, we used a MATLAB script (available on the Open Science Framework at https://osf.io/djmv8/) to numerically perform these integrations and derivatives for magnetization calculated following the Jiles-Atherton model. Note this model has no analytical solution, and thus we numerically estimated the magnetizations and resulting change in entropy using a MATLAB script. At values near saturation the Jiles-Atherton model becomes sensitive to rounding errors, and the symbolic toolbox becomes highly computationally intensive. To avoid these time intensive computations,

we performed regression analysis to approximate the relationship between the heat generation calculated by the integrals above and the difference in magnetization along the easy and hard axes. For a fixed value of KV and B, below saturation; the heat generation from the RME 1) scales linearly with the difference in magnetization ΔM, and 2) scales with the square of the magnetic moment mu_B^2. As a result, the expression for heat generated under rotation is log-linear with respect to magnetic moment. This approximation is accurate to within 1% below saturation, which accounts for all the identified biogenic materials at physiological temperatures (Fig B).

Because all of the materials we considered are below saturation in the Earth's magnetic field, this scaling approach was used to generate Fig 3c. Specifically, we calculated the difference in magnetization for all matrix elements, then calculated the heat generated for all values of anisotropy energy KV for a single value of the magnetic moment μ_B. We then scaled these values following the fitted function to find the heat generated for other values of KV and μ_B.

Another approach to calculating the heat generation under rotation calculates heat based on the change in the magnetic anisotropy energy [2, 3, 4]. In this approach, the heat generation Q from the rotating magnetocaloric effect for a uniaxially anisotropic particle from angle β_i to angle β_f is given by the difference in magnetic energy (Zeeman and anisotropy) along the starting and ending orientations: $Q = \frac{1}{T}\int_{\beta_i}^{\beta_f} dE$. The maximum heat is generated when the particle rotates from field alignment with the hard axis to field alignment with the easy axis ($\beta_i = \pi/2$ and $\beta_f = 0$ for the uniaxially anisotropic particles modeled), this gives $Q = \mu B + KV(sin^2(\theta(B, \pi/2)) - sin^2(\theta(B, 0))$. We solved for $\theta(B, \beta)$ (and thus Q) by minimizing the expression for E. This alternative approach for calculating the change in entropy gives larger values of heat generated under rotation, but does not show decreased heat under rotation at high fields, where magnetization along both axes should be near saturation and thus heat generation would be expected to decrease (due to a smaller change in magnetization, and thus smaller change in entropy, under rotation). As a result,

we use a model which predicts slightly lower changes in entropy but gives the expected behavior near saturation. For comparison we calculated the expected heat generated according to the energy-based approach, and as expected, because the heat generated is larger this model predicts more candidate particles for RME receptor. The results of this alternative approach are shown in Fig C.

Detecting changes in polarity

Although magnetic senses based on the magnetocaloric effect, chemical models, or mechanical forces on superparamagnetic particles are not expected to be polarity sensitive[5, 6], these models could detect the moment of change through a transient response as shown in models in Fig D. In this work, we have used the model for calculating the effect of chemical magnetosensation laid out in Ritz et al.[5] To determine the the effect of mechanical forces we calculate the change in open probability of a channel following Markin et al. [7]

Effects of RF fields

Magnetocaloric sensitivity to radio-frequency alternating magnetic fields (RFAMFs)

There are two sources of heat generation to consider when cycling applied magnetic fields: heat generation and absorption through the magnetocaloric effect, and heat generation through hysteresis/relaxation losses. Under the magnetocaloric effect, at high frequencies the overall effect of an alternating magnetic field is expected to be the same as the effect of a single magnetization (discussed in Duret et al. [8]), and so will be minimal for weak field strengths. Therefore, RF heating from hysteresis is expected to be the primary source of heating for weak, high frequency fields. For the particles identified as candidate RME-sensors, specific loss power (SLP) values of >6 W are attainable at frequencies used in experiments showing RFAMF-induced disorientation (Fig E).

For these calculations, we have used linear response theory (LRT) to calculate the heat generated by these particles, because the fields are far below saturation strength.[9]

Magnetomechanical sensitivity to RFAMFs

While permanently magnetized particles experiencing weak, high frequency alternating magnetic fields are not expected to be able to move in time to affect channel behavior [10], sufficiently small superparamagnetic particles will be able to reorient their magnetic domains on timescales fast enough to track with the applied field. As a result, the time-averaged magnetization of superparamagnetic particles would remain parallel to each other, resulting in a net attraction (if the moments are more aligned with the vector between the particles) or repulsion (if the moments are more perpendicular to the vector between the particles). However, the magnitude of particle attraction is primarily a function of the field amplitude, with the frequency setting the maximum particle size that can track the field. Higher frequencies would require faster relaxation times, and thus smaller particles (see Fig 3B of the main text). As a result, the small particle sizes required for high frequency fields and the weak field strengths used predict that RF effects would not be noticeable in magnetomechanical magnetosensors. At frequencies above 1 MHz, which is commonly used for RFAMF experiments, a magnetite particle's radius must be smaller than $\sim$ 8.8 nm to track the AMF. Assuming a distance between particles of 2 nm and an applied field strength of 47 μT (typical for RFAMF experiments), then the change in interparticle force compared to the case of the geomagnetic field only would only be $7.05 \times 10^{-}37$ N, far below the $1.7 \times 10^{-}13$ N gating force measured in hair cell mechanoreceptors [11] or the $1 \times 10^{-}10$ N gating force that produces mechanical stimulation of TRPV4[11].

Sensitivity to rotation and field changes at high fields

As field strengths approach saturation along both easy and hard axes, the change in magnetization under rotation (and the change in magnetic entropy) will decrease, resulting in less heat generation under rotation. As a result, the magnetocaloric model of natural magnetosensation predicts the presence of not only lower limits for sensitivity to rotation under applied fields, but also upper limits, where the magnetic field will saturate the magnetic sense (Fig F). Similarly, the same increase in the strength of an applied field will result in smaller changes in magnetization for particles near saturation, so the magnetocaloric model of natural magnetosensation predicts a loss of sensitivity to changes in field strength in high applied fields (Fig F)

Supplemental Figs

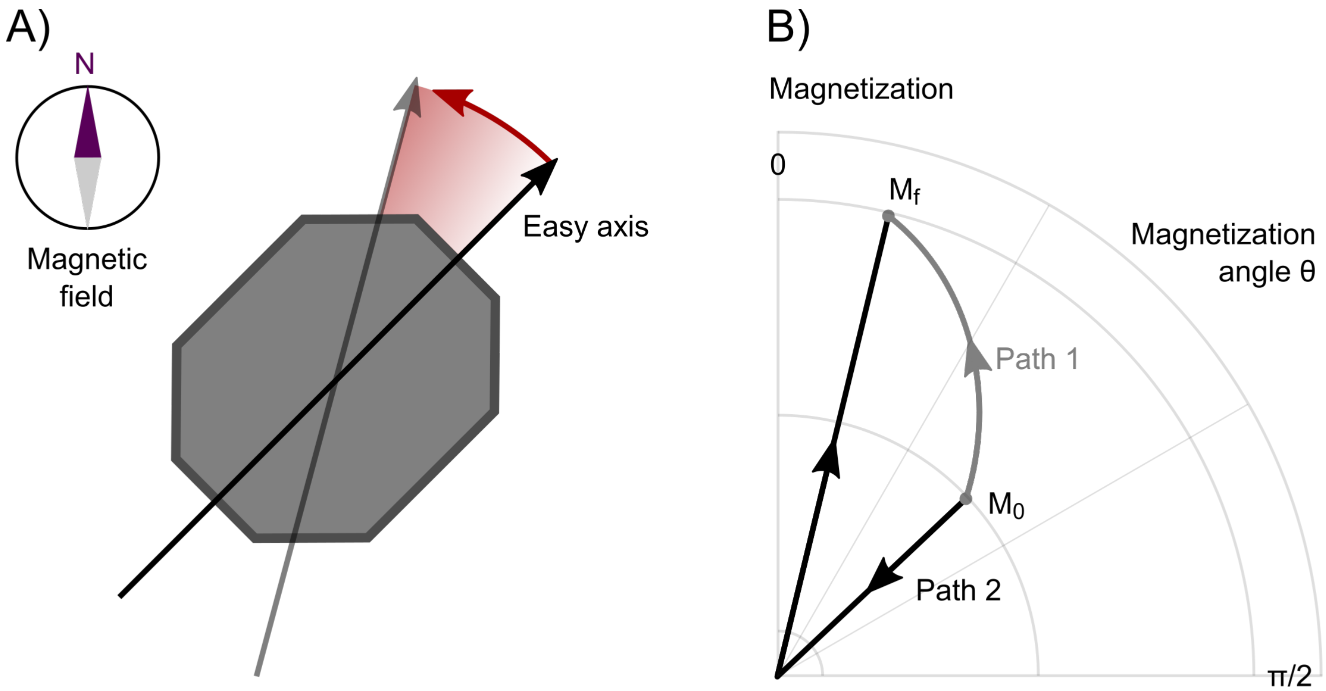

Fig A. Rotating the magnetization vector is equivalent to demagnetizing and then remagnetizing in a different direction. (A) Rotating the particle will result in a reorientation of the easy axis and corresponding change in the amplitude and direction of the (average) magnetization vector (B) The magnetization change that results from rotation (Path 1) is equivalent to removing the magnetization vector and then remagnetizing in a different orientation (Path 2).

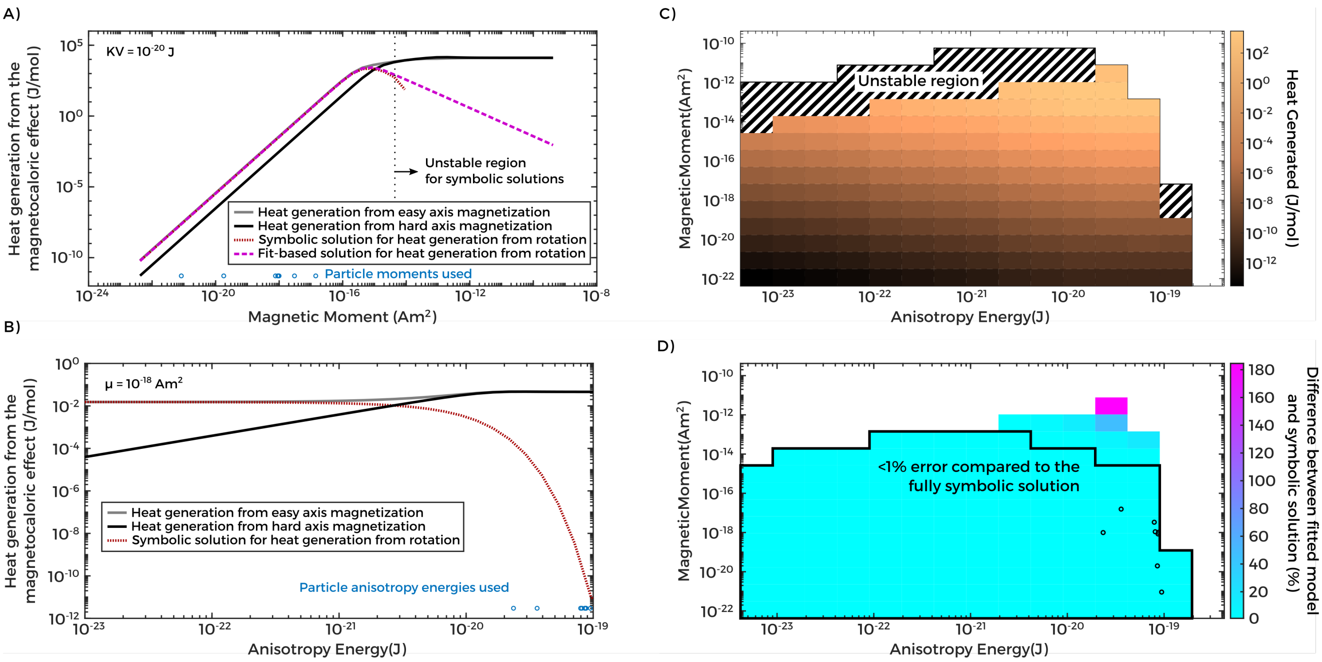

Fig B. A model based on fitting the heat generation as a function of the difference in magnetization can be used to accommodate computational constraints. (A) Below saturation, the heat generated by the magnetocaloric effect scales with the square of the particle magnetic moment (for fixed applied field, temperature, and particle anisotropy). The heat generated under rotation calculated using a MATLAB script using the symbolic toolbox (red dotted line) is given by the difference of heat generated by applying the heat along the easy (grey line) and hard (black line) axes. A model based on fitting the relationship between heat generation and the magnetization along each axis (magenta dashed line) reproduces the results of the symbolic toolbox below saturation while remaining stable beyond saturation. Note that the moments of the particles examined in this work (blue circles) are below saturation. (B) The scaling of magnetocaloric heat generation with particle anisotropy. While certain combinations of moment and anisotropy energy can produce unstable solutions (shown in C), for low moments the symbolic solver remains stable. (C) The symbolic toolbox returns invalid results for high moments and anisotropy energies. The hatched region represents elements permitted by Néel relaxation theory in which the symbolic solver returns invalid heat values. (D) A fitted model based on the difference in magnetizations matches well below saturation, including for all particles used in this study. All of the particles we examined (black circles) are in regions which show less than 1 percent difference between the fully symbolic solver and the fitted model (outlined in black).

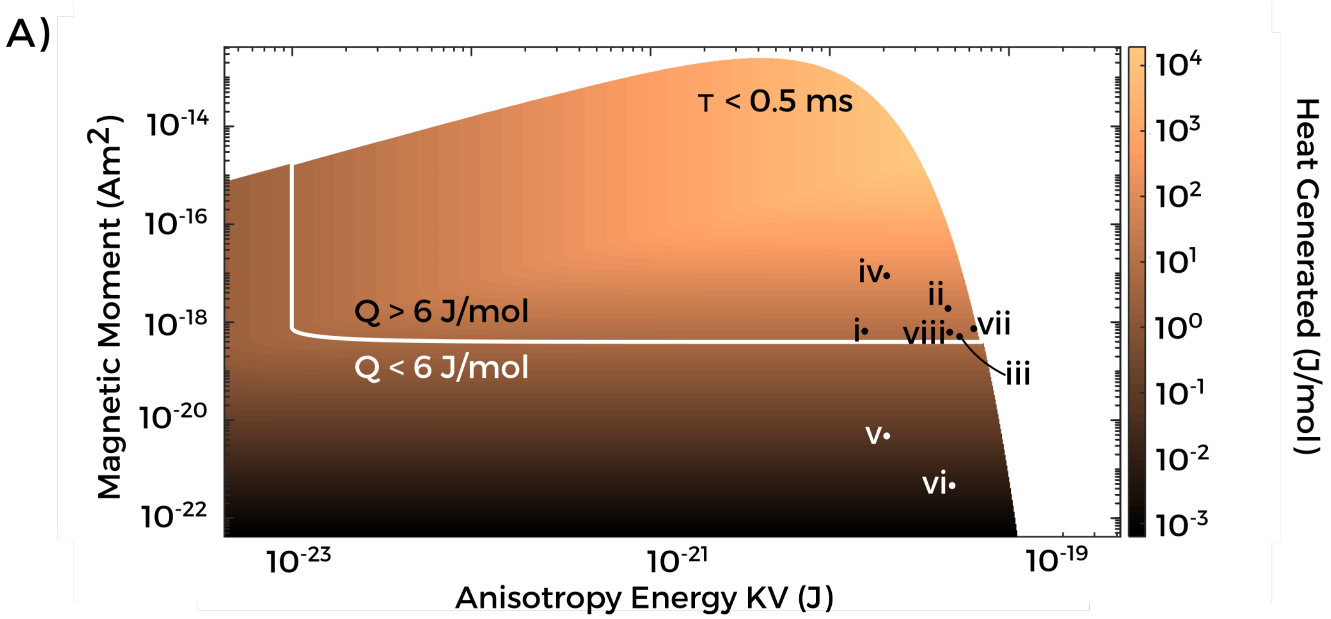

i	7 nm magnetite	ii	10 nm magnetite
iii	17 nm maghemite	iv	100 nm hematite
v	5 nm ferrihydrite	vi	1 nm wüstite
vii	9 nm greigite	viii	50 nm goethite

Fig C. An alternative model of magnetocaloric heat generation predicts larger heat generation under rotation. This heatmap shows predicted heat generation from rotation from hard axis alignment to easy axis alignment in an applied 25 μT field. This model predicts higher heat generation and thus more candidate particles would be expected to produce at least 6 J/mol. Note that this model does not show the reduction in heat generation at high moments that would be expected from the magnetization along both axes nearing saturation.

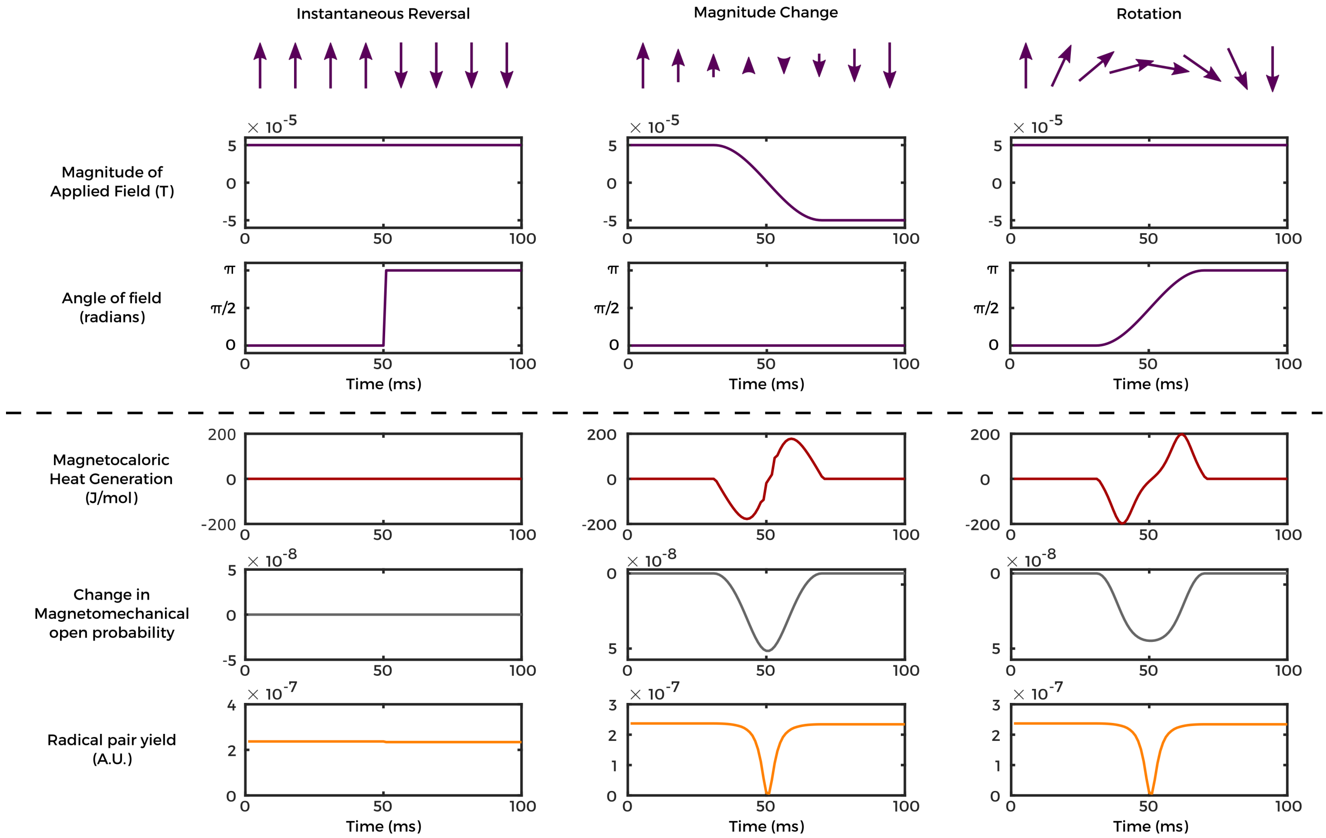

Fig D. Even polarity insensitive mechanisms may be able to detect polarity reversals. Three cases of polarity reversal of a 50 μT field are shown (columns), for each of three polarity-insensitive magnetosensation models (rows below dashed line). The magnetosensation models are: the magnetocaloric effect, showing the heat generation from the change in applied field for a 12 nm radius magnetite; a magnetomechanical model based on two of the same superparamagnetic particles 3 nm apart and both attached to a TRPV4 channel; and a radical pair (chemical) model based on simple isotropic hyperfine coupling of 500 μT. For an instantaneous reversal in field polarity, none of the models are expected to detect the reversal, whereas for slower changes, whether from varying field magnitude or direction, each of the senses may show a transient effect.

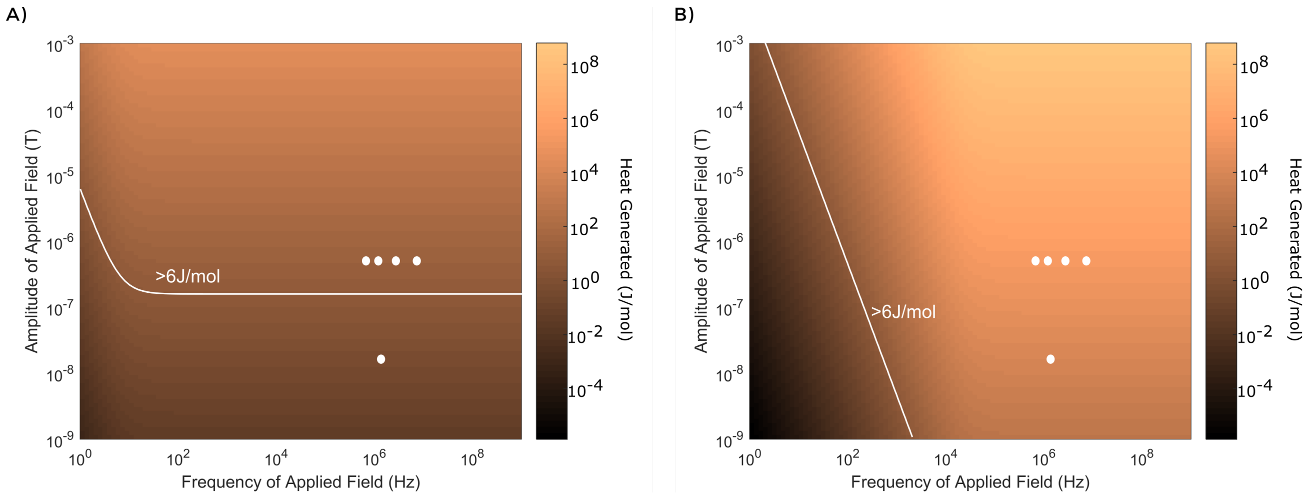

Fig E. Many of the RF experiments shown to disrupt orientation ability would be expected to produce several Watts of heat in good candidates for an RME-sensor. In (A) 12 nm magnetite and (B) 120 nm hematite, linear response theory models predict that even sub-microtesla fields can generate appreciable levels of heat, above 6 J/mol per second (6 W). Note that lower frequencies produce less heating, and so may account for the lack of disorientation seen in experiments for lower frequency fields.

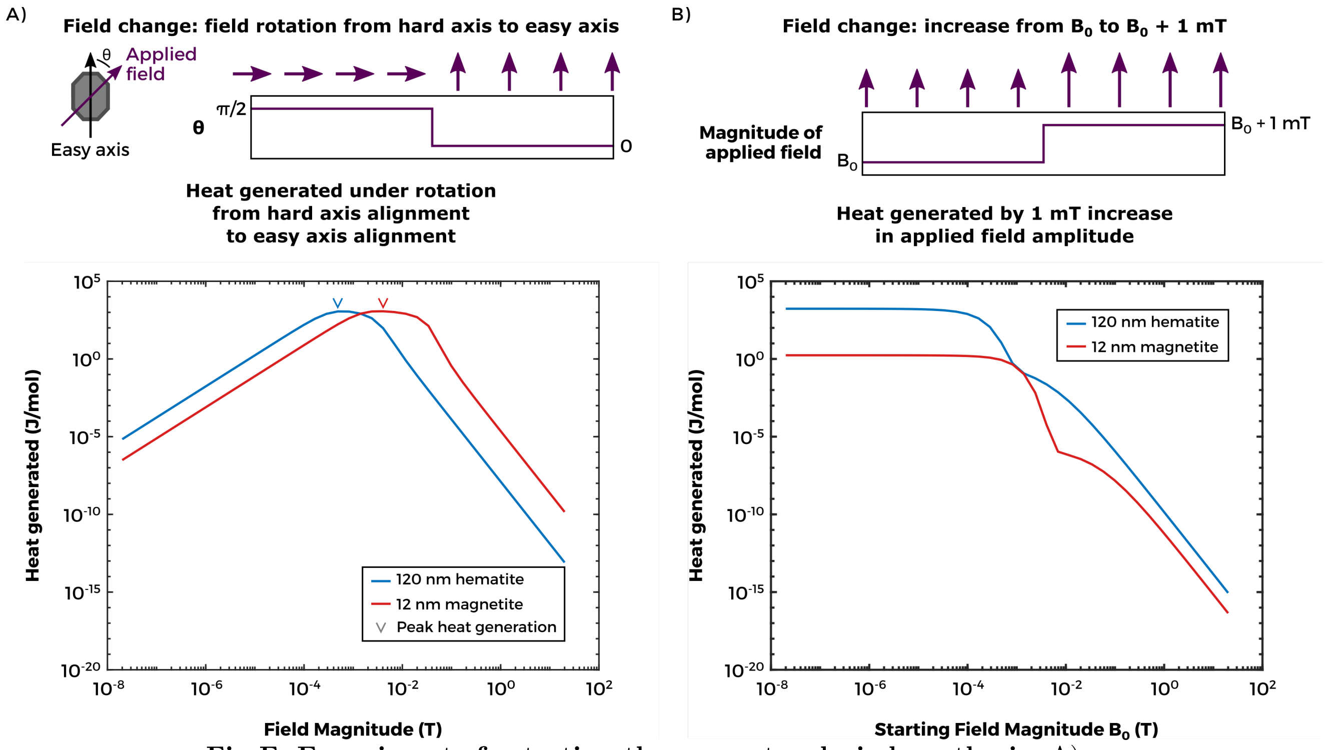

Fig F. Experiments for testing the magnetocaloric hypothesis. A) RME-sensors are expected to become less sensitive to rotation at high fields. The magnetocaloric model predicts that heat generation under rotation will increase with increasing field strength to a peak value before decreasing as the field begins to achieve saturation magnetization levels along both the easy and hard axes. In the case of 12 nm radius magnetite, this model predicts the peak heat generation under rotation at 300K will occur near 4 mT, while in 120 nm hematite it predicts maximum heat generation near 0.5 mT. B) Magnetocaloric sensors are expected to become insensitive to step changes in field strength at high field strengths. The heat generated by a small increase in applied field is expected to decrease at higher starting fields, because the particle's starting magnetization nears saturation. Near saturation, the same increase in applied field will result in a smaller increase in magnetization, and correspondingly the magnetocaloric effect will generate less heat. Lines are 12 nm magnetite (red) and 120nm hematite (blue)

References

1. de Oliveira NA, von Ranke PJ. Theoretical aspects of the magnetocaloric effect. Physics Reports. 2010;489(4-5):89–159. doi:10.1016/j.physrep.2009.12.006.

2. Balli M, Jandl S, Fournier P, Dimitrov DZ. Giant rotating magnetocaloric effect at low magnetic fields in multiferroic TbMn 2 O 5 single crystals. Applied Physics Letters. 2016;108(10):102401. doi:10.1063/1.4943109.

3. Zhang XQ, Wu YD, Ma Y, Dong QY, Ke YJ, Cheng ZH. Large rotating magnetocaloric effect in ErAlO 3 single crystal. AIP Advances. 2017;7(5):056418. doi:10.1063/1.4974915.

4. Nikitin SA, Skokov KP, Koshkid'ko YS, Pastushenkov YG, Ivanova TI. Giant Rotating Magnetocaloric Effect in the Region of Spin-Reorientation Transition in the NdCo5 Single Crystal. Physical Review Letters. 2010;105(13):137205. doi:10.1103/PhysRevLett.105.137205.

5. Ritz T, Adem S, Schulten K. A model for photoreceptor-based magnetoreception in birds. Biophysical Journal. 2000;78(2):707–718. doi:10.1016/S0006-3495(00)76629-X.

6. Davila AF, Fleissner G, Winklhofer M, Petersen N. A new model for a magnetoreceptor in homing pigeons based on interacting clusters of superparamagnetic magnetite. Physics and Chemistry of the Earth, Parts A/B/C. 2003;28(16-19):647–652. doi:10.1016/S1474-7065(03)00118-9.

7. Markin VS, Sachs F. Thermodynamics of mechanosensitivity. Physical Biology. 2004;1(2):110–124. doi:10.1088/1478-3967/1/2/007.

8. Duret G, Polali S, Anderson ED, Bell AM, Tzouanas CN, Avants BW, et al. Magnetic entropy as a proposed gating mechanism for magnetogenetic ion channels. Biophysical Journal. 2019; p. 148379. doi:10.1016/j.bpj.2019.01.003.

9. Harabech M, Leliaert J, Coene A, Crevecoeur G, Van Roost D, Dupré L. The effect of the magnetic nanoparticle's size dependence of the relaxation time constant on the specific loss power of magnetic nanoparticle hyperthermia. Journal of Magnetism and Magnetic Materials. 2017;426(November 2016):206–210. doi:10.1016/j.jmmm.2016.11.079.

10. Hore PJ, Mouritsen H. The Radical-Pair Mechanism of Magnetoreception. Annual Review of Biophysics. 2016;45(1):299–344. doi:10.1146/annurev-biophys-032116-094545.

11. Matthews BD, Thodeti CK, Tytell JD, Mammoto A, Overby DR, Ingber DE. Ultra-rapid activation of TRPV4 ion channels by mechanical forces applied to cell surface $\beta1$ integrins. Integrative Biology. 2010;2(9):435. doi:10.1039/c0ib00034e.
